# Axonal mRNA translation in neurological disorders

**DOI:** 10.1080/15476286.2020.1822638

**Published:** 2020-09-29

**Authors:** Julie Qiaojin Lin, Francesca W. van Tartwijk, Christine E. Holt

**Affiliations:** a UK Dementia Research Institute at University of Cambridge, Department of Clinical Neurosciences, Island Research Building, Cambridge Biomedical Campus, Cambridge, UK; b Department of Chemical Engineering and Biotechnology, University of Cambridge, Cambridge, UK; c Department of Physiology, Development and Neuroscience, University of Cambridge, Cambridge, UK

**Keywords:** Neurological disorders, local protein synthesis, RNA-binding protein, axonal trafficking, axon branching, axon survival, neuronal stress

## Abstract

It is increasingly recognized that local protein synthesis (LPS) contributes to fundamental aspects of axon biology, in both developing and mature neurons. Mutations in RNA-binding proteins (RBPs), as central players in LPS, and other proteins affecting RNA localization and translation are associated with a range of neurological disorders, suggesting disruption of LPS may be of pathological significance. In this review, we substantiate this hypothesis by examining the link between LPS and key axonal processes, and the implicated pathophysiological consequences of dysregulated LPS. First, we describe how the length and autonomy of axons result in an exceptional reliance on LPS. We next discuss the roles of LPS in maintaining axonal structural and functional polarity and axonal trafficking. We then consider how LPS facilitates the establishment of neuronal connectivity through regulation of axonal branching and pruning, how it mediates axonal survival into adulthood and its involvement in neuronal stress responses.

## Introduction

The nervous system is an interconnected network of billions of individual cells, which is key to its function. As central network building blocks, neurons not only conduct signals to relay information (electrically within and chemically between cells), but also generate, maintain, and adapt inter-neuronal connections to enable dynamic information storage and retrieval (i.e., memory and learning). The sites of connection, synapses or neuroeffector junctions, where the axon terminal of one neuron meets the dendritic spine or soma of another neuron or a target cell, are key for cognition, as well as for control and coordination of the body [1,2]. Aberrant network assembly or progressive network disintegration, due to failure in the establishment or maintenance of synaptic connections, results in neurodevelopmental and neurodegenerative disorders, respectively.

In this review, we focus on the idea that the local synthesis of new proteins (local protein synthesis; LPS) in axons by translation of localized mRNAs is essential for network assembly and its maintenance in adulthood. Evidence that axons can synthesize proteins locally was first reported in axons in the 1960s using metabolic labelling methods [3,4,5], but has only become widely accepted in recent years. Early scepticism sprang from concerns about sample (axonal) purity due to technical difficulties in obtaining axon-only material, and the paucity of ultrastructural evidence for the existence of ribosomes in axons. Technical advances in recent years have overcome these difficulties, enabling the collection of pure axons *in vitro* [6,7], the use of sophisticated RNA molecular analysis (transcriptomics and translatomics) [8–10] and the acquisition of ultrastructural evidence of ribosome localization in axons [9,11,12,13]. As a consequence, evidence now abounds that thousands of diverse sets of mRNAs reside and are translated in axons of both central nervous system (CNS) and peripheral nervous system (PNS) neurons. However, the exact contribution of axonal translation to function *in vivo* has been slow to emerge due to the scarcity of approaches that enable precise and controlled inhibition of protein synthesis in axons without affecting cell bodies. The first *in vivo* experiment where the axonal translation of a specific mRNA was blocked was done in the *Xenopus* vertebrate visual system [14]. Remarkably, without the translation of a specific intermediate filament protein (Lamin B2), the retinal axons degenerated; hence, the notion that LPS was needed for axon maintenance was born. It is now known that the axonal transcriptome consists of several groups of mRNAs with related functions, which are bound by particular RNA-binding proteins (RBPs) [15]. Meanwhile, research on proteins associated with neurodegenerative diseases has identified an increasing number of disease-associated RBPs, such as Fused in Sarcoma (FUS) and Survival of Motor Neuron (SMN) [16,17,18], providing a parallel strand of evidence linking axon health to RNA regulation. The role of four of these disease-associated RBPs, namely FUS, SMN, Fragile-X Mental Retardation Protein (FMRP), and TAR DNA-binding protein 43 (TDP-43), in local translation in axons and dendrites has recently been reviewed [19]. Here, we discuss the intertwining strands of research on axonal LPS and RBP dysregulation, and in particular, explore the relevance of their combined findings to neurological disorders. We focus on neurological disorders with genetic components, examining to what extent the genetic alterations associated with these diseases (in RBPs as well as other proteins) support a causative role of LPS in pathogenesis or disease progression.

### Long-term neural networks rely on cellular specializations

In this section, we briefly examine some specialized features of neurons that underpin neural network assembly and function, particularly the subcellular processes crucial for the *in vivo* development and maintenance of neuronal processes – dendrites and axons – which, collectively, we refer to here as ‘neurites’. In subsequent sections, we discuss how some of these requirements are met by LPS.

The formation of a large number of synaptic connections between cells with cell bodies that may be far apart requires neurons to be exceptionally structurally and functionally *polarized*. The average human neocortical neuron forms around seven thousand different synapses with multiple different cells [20], and each synaptic cleft has to be narrow enough to allow rapid and specific signal transmission relying on neurotransmitter diffusion, which results in a breadth of around 20 nm in the central nervous system [21]. Such spatial organization can only be possible if neurons are morphologically polarized: neurons extend long and sometimes branched axons towards the soma or highly branched dendrites of recipient neurons. Axons in particular can reach great lengths, with the longest in the human body being those of motor neurons (up to one meter in length). This length has two further consequences: it limits the speed of macromolecule exchange between axon terminals and the soma, and it places distal parts of axons in different local environments than the soma. Therefore, axons require (i) an efficient active transport mechanism to achieve a stable supply of locally required factors (including mRNAs, proteins, and organelles), which must function efficiently in the spatially confined environment of elongated axon. In practice, the fastest axonal transport mechanisms can reach speeds of around 400 mm/day [22], which is much faster than passive diffusion (especially for molecules with diameters of more than 40 nm, for which the diffusion coefficient drops below 1 μm^2^/s in nerve cytoplasm [23]). Furthermore, as distal axons can experience very different stimuli than the soma, they need (ii) the ability to independently remodel or change their macromolecular components.

To achieve almost immediate information relay from dendrites to axons at a speed beyond what can be reached by active transport, neurons are electrically *excitable*. In order for information to be transferred between cells, even fast axonal transport is insufficient: when a hand is withdrawn reflexively from a hot surface, for instance, a signal must travel from the hand to the spinal cord and back to relevant muscles, which is well over a meter of total path length and so would take several days by active transport [22]. In contrast, the unidirectional transmission of changes in membrane potential (action potentials) along axons can reach speeds of over 100 m/s [24], and so can accomplish this information transfer in well under a second. However, excitability comes at an energetic cost. The restoration of dissipated ion gradients following action potentials accounts for the majority of the large neuronal energy expenditure on signalling [25]: it has been estimated that three-quarters of neuronal energy consumption are spent on signalling [26], which is not trivial, considering the central nervous system accounts for 20% of the human body’s energy consumption, but for only 2% of its weight [27]. In addition to membrane potential management, this high energy consumption is accounted for by vesicle recycling, neurotransmitter synthesis, and axonal transport [28]. Therefore, another requirement for neuronal function arises, namely that (iii) high energy consumption must be supported throughout neurites. This requires the continual presence of a population of mitochondria in neurites.

In order for neuronal networks to learn, they must be able to *adapt* the nature of connections according to various stimuli, as changes in synaptic strength (plasticity) are thought to be important for (efficient) learning and memory [2,29]. This is one of the ways in which neuritic (sub)compartments need to be able to locally change their macromolecular components (ii): as part of synaptic plasticity, components should be changed to alter local synaptic function in response to changes in activity. Furthermore, neurons should be able to add new connections, reduce unused connections, and remove damaged connections. Therefore, synaptic structural plasticity calls for (iv) tightly regulated *local* ‘death-like’ pathways to remove synapses and even whole axons, as well as for mechanisms to add new synapses.

Lastly, for neuronal networks to store memories long-term, neurons have to be *resilient* against a range of insults, in order to sustain neural connectivity throughout the organism’s life span. Consequently, neurons are long-lived cells, particularly in comparison with other cell types, such as the intestinal epithelium or red blood cells, which are frequently ‘worn out’ and replenished by reservoirs of stem cells. However, neurons cannot be similarly replaced, as new neurons could not readily integrate into the neuronal network without loss of the information encoded by pre-existing synaptic connections. Notably, adult neurogenesis and subsequent integration of newly formed neurons do in fact occur in the mammalian brain, but only in the olfactory bulb and dentate granule cell layer of the hippocampus, in a process that is modulated by circuit activity [30]. Therefore, the following is required to appropriately maintain neuronal networks: (v) neuronal stress responses should adopt anti-apoptotic strategies to enhance stress tolerance and to avoid cell death, and (vi) neurons must habituate to and mitigate cellular damage accumulated during aging. These unique stress responses have to affect local processes in neurites, including local replenishment and activation of anti-stress factors that involve LPS and post-translational modifications (PTMs), which also become altered with age.

### LPS supports multiple axonal functions

LPS enables neurites to autonomously remodel their proteome in response to local stimuli, which means it can provide a way to address some of the requirements outlined above. This is particularly true for the axon [31], which is the longest neurite and contains the largest cytoplasmic volume of any compartment of the mature neuron [32,33].

LPS can be useful to maintain local axonal proteome homeostasis, but its products may also have unique properties that carry functional information. These can arise from their association with local components of signalling cascades or from unique post-translational modifications [34]. For instance, a study in cultured primary hippocampal neurons showed locally produced arginyltransferase 1 (ATE) in the growth cone arginylated adjacent β-actin proteins that were also locally synthesized, and that the arginylation of β-actin in neurites is important for growth cone area size (spreading) and neurite outgrowth [35].

A wide range of mRNAs has been demonstrated to be locally translated, which contribute to a variety of subcellular functions and neuronal specializations beyond synaptic plasticity. In the axon, locally synthesized proteins have been shown to contribute to axon navigation, maintenance and regeneration [36]. Specifically, LPS regulates a range of essential processes in the axon [31], including vesicle trafficking, cytoskeletal remodelling and mitochondrial integrity [37].

**Figure 1.**
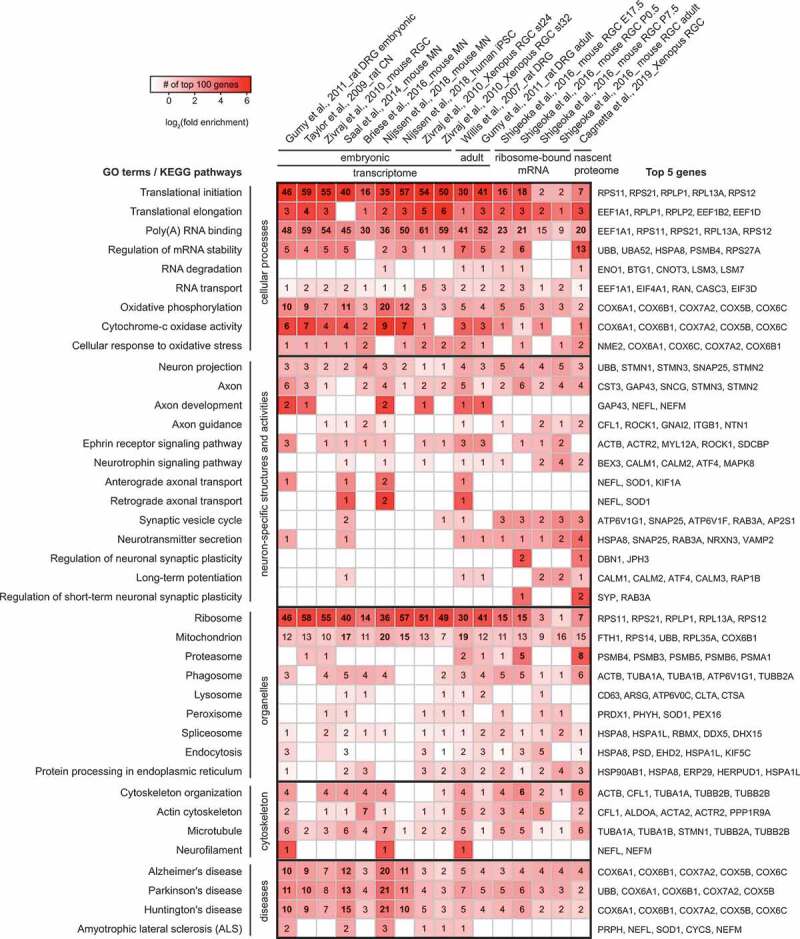
Selective GO terms and KEGG pathways in most abundant axonal transcripts, ribosome-bound mRNAs and nascent proteins

Notably, the translatome is not static, which allows it to support a range of functions. Genome-wide analyses have revealed that the axonal translatome changes during the course of development, in step with evolving axon function and behaviour. In mouse retinal ganglion cell (RGC) axons *in vivo*, for example, the mRNAs translated in early growth stages are associated with axon elongation, followed by branching then synaptogenesis [9]. The context-dependent composition of the axonal translatome is further demonstrated by functional enrichment Gene Ontology (GO) and KEGG pathway analyses of published datasets describing the abundant localized mRNAs and locally synthesized proteins in axons at different developmental stages in different neuronal types [8–10,38–43] (Fig. 1). mRNAs of ribosomal proteins are highly enriched in axons of all stages, as reported by several studies [9,10,40,44]. However, only a subset is bound to ribosomes, according to an axon-TRAP study, and their translation rates decline synchronously after the axonal branching stage [9]. It has been further demonstrated that several ribosomal proteins, particularly the surface components of each subunit, are locally synthesized upon cue stimulation and incorporated on-site into axonal ribosomes [44]. The functional role of this axonal ribosome remodelling is not yet known, but it could extend the lifetime of ribosomes and, perhaps most intriguingly, could ‘tune’ them to translate specific mRNAs [45].

Top 100 annotated genes with most axonal reads in 16 datasets from 9 independent studies (4 microarray, 3 RNA-Seq, 1 Ribo-Seq and 1 nascent proteomic studies) are included in this analysis. The heat map shows the enrichment of GO terms and KEGG pathways relevant to the discussion in this review. The colours of the heat map represent the log2 value of the fold enrichment. The numbers on the heat map indicate the total number of genes among the top 100 genes from each dataset associated with the GO term/KEGG pathway and those with a Benjamini-Hochberg value <0.05 are shown in bold. Human orthologs of the top 2–5 genes associated with each GO/KEGG category ranked by their appearance frequency are indicated next to each row. The enrichment analysis was carried out with DAVID v6.8.

In addition to ribosomal proteins, axonal localization and translation of mRNAs encoding other proteins with roles in LPS are also revealed by the analyses, including those regulating mRNA metabolism (e.g., ubiquitin and proteasome components), those transporting and localizing mRNA (e.g., cytoskeletal proteins and RBPs), those forming part of the translation machinery (e.g., eukaryotic initiation and elongation factors), and those required for energy supply (e.g., mitochondrial proteins). In addition, though mRNAs encoding synaptic components are not strongly enriched, these proteins, including synaptosomal-associated protein 25 (SNAP25) and vesicle-associated membrane protein 2 (VAMP2), are more abundant in the local translatome [9,39]. Furthermore, some components of the oxidative stress response may be locally synthesized to respond to local perturbations of energy supply and mitochondrial function.

Besides housekeeping proteins produced via basal translation (Fig. 1), the stimulus-dependent translatome is also a large constituent of axonal proteome. Stimulus-dependent LPS contributes to a range of axonal functions: it mediates axon guidance and arborization, supports axon maintenance and survival, regulates presynapse formation and synaptic plasticity, and aids the response to stress and injury [31,46,47]. During axon pathfinding in development, asymmetric localization and translation of *β-actin* mRNAs in the growth cone can be observed in cultured *Xenopus* RGCs upon 5–10 min gradient stimulation with the guidance cue Netrin-1 or brain-derived neurotrophic factor (BDNF), which facilitates growth cone turning [48,49]. As detected by metabolic labelling, 1-h cue stimulation of developing RGC axons induced a 10–80% increase in the amount of locally synthesized proteins [14]. A recent proteomic study of axonal nascent proteome showed that among 1000 proteins detected in isolated axons, approximately 350 proteins were locally synthesized. The translation rate of over 100 of them changed significantly upon guidance cue stimulation and the pattern of changes varied greatly depending on the types of the cues and lengths of stimulation [39]. In mature neurons, LPS can provide a basis for heterogeneity of synapses made by the same neuron: for instance, LPS enables the activity-mediated upregulation of the key presynaptic kinase CamKII in the *Drosophila* larval neuromuscular junction [50]. In the model system of *Aplysia* sensory-motor neuron synapses, presynaptic LPS has been shown to support synaptic plasticity: branch-specific long-term facilitation in response to localized exposure of serotonin requires presynaptic LPS [51], for instance of the peptide neurotransmitter sensorin [52]. Moreover, different aversive stimuli, including acute injury or chronic diseases, elicit distinct landscapes of the local translatome, opening up new opportunities to discover therapeutic targets [8,42,47,53].

### RBP dysfunction in neurological disorders indicates compromised LPS may be causative

**Figure 2.**
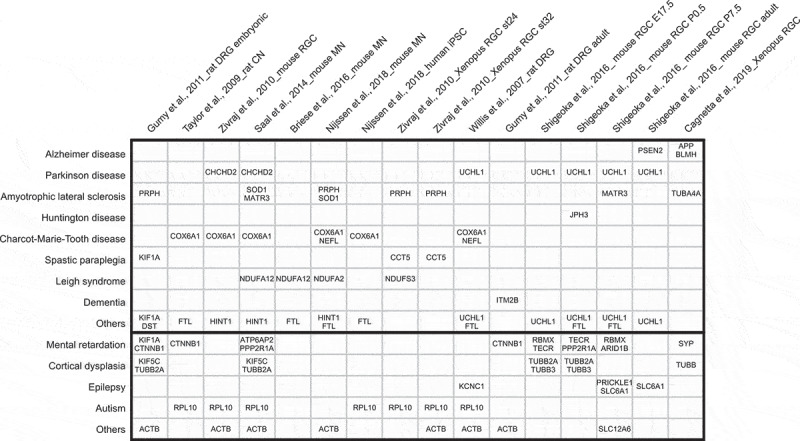
Disease-associated genes enriched in axonal transcriptomes and translatomes

Considering the range of critical processes in which LPS is involved in neurons, including in axons, it is not surprising that it is disturbed in multiple neurological disorders, and that this disturbance may be part of the pathomechanism(s) of these disorders. Indeed, a bioinformatics search among the highly abundant axonally localized or translated mRNAs identifies a number of genes associated with various neurological disorders (Fig. 2), including amyloid *β* precursor protein (APP) and ubiquitin carboxyl-terminal hydrolase L1 (UCHL1) related to Alzheimer’s disease (AD) and Parkinson’s disease (PD) susceptibility [9,39,43]. ‘Neurological disorder’ is a broad term referring to any condition in which the function of CNS and/or PNS deteriorates. It covers a wide range of diseases, which place a significant burden on patients and society: neurodevelopmental disorders such as Fragile X syndrome (FXS), autism spectrum disorder (ASD), and schizophrenia, neurodegenerative disorders like AD, PD, and amyotrophic lateral sclerosis (ALS), and acquired disorders, addictions, and injury- or pathogen-induced disorders. Familial neurological disorders are associated with highly or completely penetrant mutations, which can be used not only to develop *in vitro* or *in vivo* disease models, but also link the disease to perturbations of certain cellular processes.

A table shows human orthologs of axonally enriched transcripts or nascent proteins dysregulated in common neurodegenerative or neurodevelopmental diseases among the 100 most abundant genes in each dataset. Dysfunction of the indicated genes either causes or increases susceptibility to the disease, based on the corresponding OMIM disease entries.

Interestingly, structural and functional alterations of RBPs are implicated in neurodevelopmental and neurodegenerative disorders, which strongly points to dysregulation of gene expression as a key feature of diseases. For instance, FXS is caused by loss-of-function mutations in the neuronal RBP FMRP [54]. However, for many neurological disorders in which RBPs can be found mutated, the genetic basis of familial disease variants is less readily interpreted than for FXS.

The case of ALS illustrates the two main reasons why genetic predisposition of a disease does not always readily lead to a hypothesis of pathogenesis [55]. Firstly, the genetic basis of familial ALS (fALS) is heterogeneous. Mutations of genes encoding the RBPs FUS and TDP-43 are prevalent among fALS cases. Since RBPs are key to localization of mRNAs and the regulation of translation, their altered function has, in some cases, been linked to the perturbation of LPS in axons [18,56–59]. However, highly penetrant mutations have also been discovered in other genes, such as in those encoding the following proteins: C9orf72 [60,61], the antioxidant enzyme superoxide dismutase (SOD1) [62], the motor protein kinesin heavy chain isoform 5A (KIF5A) [63], tubulin isoform alpha 4A (TUBA4A) [64], and the actin-associated protein profilin 1 [65]. Secondly, mutant proteins can be expressed in (or lost from) a range of cell types, but the disease phenotype appears restricted to nervous tissues or even certain types of neurons. For instance, FUS and TDP-43 are ubiquitously expressed in all cells [66,67], but their mutations do not affect all tissues or even all neuronal subtypes. Though motor neurons are primarily affected, the extent of degeneration of different motor neuron subtypes varies greatly, with for instance spinal cord motor neurons degenerating relatively early in disease and ocular motor neurons remaining unaffected up to the end stage of the disease [68,69]. However, it should be noted that though the diagnosis of ALS is based on motor symptoms, ALS is increasingly recognized to be associated with a range of non-motor phenotypes in patients: for instance, up to half of ALS patients display some form of cognitive impairment, with 15% meeting the criteria for frontotemporal dementia (FTD) [70]. In fact, ALS shares many pathological features as well as genetic risk factors with frontotemporal dementia (FTD), which like ALS is associated with mutations in and aggregates of TDP-43, and these diseases are considered to be part of the same ‘disease continuum’ of TDP-43 proteinopathies [69]. In such co-occurring ALS/FTD, non-motor neuronal subtypes are also affected: TDP-43 inclusions have been identified in the cortex and hippocampus of both sporadic and C9orf72-associated ALS/FTD patients [71].

Then, the postulation that RBP dysfunction can be causative in multiple neurological disorders, such as ALS, leaves two unanswered questions. Firstly, why do certain mutations in widely expressed RBPs such as FUS exert particularly strong effects on neurons? Secondly, why does RBP dysfunction result in the same phenotype as mutations of other disease-related proteins, such as cytoskeleton-associated proteins?

To begin to answer these questions, the functions of RBPs in neurons require further consideration. Typically, an individual RBP is functionally versatile and some of these functions may be unique to neurons (e.g., due to the presence of neuronally expressed interaction partners). Alternatively, the RBP’s functions may be exceptionally important in neurons. Neuropathology caused by RBP loss-of-function mutations indicates the protein performs an essential role on which neurons rely, whereas for a gain-of-function mutation (such as aggregation), the neuron would be particularly sensitive to this effect. The latter is best illustrated by proposed pathogenesis of neurodegenerative disorders: accumulation of protein deposits containing RBPs is a hallmark of multiple neurodegenerative disorders, such as FUS and TDP-43 aggregates in ALS [72,73,74,75]. Meanwhile, loss-of-function models have also been put forward: functional loss of FUS may affect mRNA stability at dendritic spines and cause axonal transport defects [76]. Therefore, there has been a long debate whether the pathological aggregate is in itself toxic, or whether loss of RBP function is detrimental. However, recent advances in genetic and pathophysiological studies suggest the two theories are not mutually exclusive and their distinction may be blurred, as heterogenous genetics can sometimes converge to shared downstream effects observed in a disease, such as impaired synaptic connectivity.

In the following sections, we provide a summary of evidence and our speculations on how functional alteration of RBPs and other disease-associated proteins may lead to LPS dysregulation in neurites. Using key cellular processes in axonal compartments as examples, we examine potential links between aberrant LPS and observed phenotypes of common neurological disorders, and propose that LPS may serve as a crucial mediator in neuronal health and viability.

## Polarity and axonal trafficking

The length and narrowness of axons create specific physical challenges for the transport of cargos, including mRNAs and translational machinery as well as organelles and proteins. Firstly, the narrowness of axons largely limits the distribution of materials by simple diffusion, as it affects flow – the diameters (calibres) of adult axons are typically between 0.1–1 µm for unmyelinated axons [77]. According to Stokes’ law, the opposing force impeding an object’s motion in a viscous fluid is proportional to the object’s size, the fluidic viscosity, and the flow velocity. However, boundary effects (a reduction in flow velocity as fluids approach the wall) play a much more significant role in a narrow cylindrical geometry than a large space (such as a cell body). Therefore, moving cargos encounter greater opposing forces within axons than within the soma, where most of the molecules are relatively far from the plasma membrane [78]. This is best demonstrated by comparing the speed of fast axonal transport (2–5 µm/s) [79] and diffusion coefficient of a GFP molecule in the cytoplasm (7.7–126 µm^2^/s) [80,81,82]. The second challenge to axonal cargo trafficking is posed by local macromolecular crowding in the axoplasm, which is packed with a dense cytoskeletal network and both static and moving cargos. For instance, membrane-bound and membraneless organelles in axons range from 100 nm to 1–2 µm in diameter, which is close to the average axon calibre of around 1 µm [77]. Local crowded regions in axons may act as physical barriers, resulting in a decrease of cargo velocity or complete stalling.

As a consequence of this limited diffusion, neurons have evolved unique strategies to facilitate the interlinked processes of RNA localization, local translation and axonal transport. These include the establishment of a robust scaffold to maintain axon morphology, and of an active transport network that can counteract drag forces and respond to changes in crowdedness [83,84]. Cytoskeletal elements, motor proteins and adaptor proteins together form the basis of these structures. In addition, RBPs are key for axonal RNA transport through interaction with motor and adaptor proteins. It is now clear that disruption of axonal transport is closely associated with multiple neurological disorders [85,86,87], as are structural and functional impairments of the main axonal cytoskeletal elements [83,87,88].

**Figure 3.**
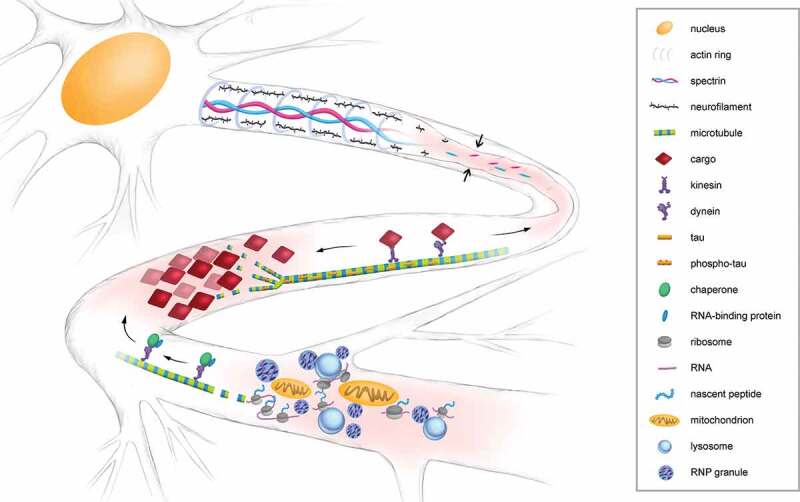
**Mechanisms to sustain axonal transport related to LPS**. Neurofilaments and membrane-associated periodic skeleton regulate axon structure (upper segment); microtubule and motor protein-based active transport maintains cargo trafficking (middle segment); modulation of axonal RBP, RNA and organelle density controls local macromolecular crowdedness (lower segment). Perturbation of these processes can result in defective axonal trafficking, as indicated by pink axon segments

In this section, we discuss some of the cytoskeleton-related processes compromised in diseased neurons, dysregulation of which results in errors in mRNA localization and therefore LPS (Fig. 3). Interestingly, the interaction between LPS and axonal transport can at times be bidirectional, as a number of studies have revealed axonal localization of mRNAs encoding cytoskeletal building blocks (i.e., neurofilament proteins, β-actin, tubulins) and their associated proteins (e.g., RhoA, cofilin, tau), some of which have been shown to be locally translated [89]. Impaired local synthesis of these cytoskeletal components and modulators would be expected to lead to disrupted axonal trafficking and/or disease progression. However, the concept of a direct link between axonal expression of cytoskeletal proteins and pathogenesis of neurological disorders remains largely hypothetical. To explore this hypothesis, we will next highlight some cytoskeletal components suggested to be locally synthesized.

### The axonal cytoskeleton maintains axon structure and organization

To maintain structural and functional polarity and sustain transport of cargos of various sizes, it is important that axons are mechanically resilient: axon shafts do not collapse around their circumferences or break during axon elongation or upon deformation by surrounding cells and tissues [90]. The axon diameter is mainly regulated by neurofilaments and actin filaments [91]. Currently, the correlation between axon calibre and neuronal vulnerability in neurodegeneration is still controversial [92], but retaining axonal radial structure and elasticity is undoubtedly important for intra-axonal trafficking and therefore LPS.

Neurofilaments are a type of intermediate filaments most abundant in axon shafts, which structure and organize axons in several ways. Firstly, they are a major determinant of axon calibre, particularly for large axons: a large axon diameter is often associated with a large number of axonal neurofilaments and increased inter-neurofilament spacing [93,94], and loss of neurofilaments results in a reduction in axon calibre and conduction velocity, leading to impairments in axon development, survival, and regeneration [95]. Secondly, neurofilaments interact with axonal organelles and cytoskeletal components. For instance, neurofilaments serve as scaffolds for docking and positioning of endoplasmic reticulum (ER), endosomes, mitochondria and synaptic vesicles in axons [96]. One study in cultured dorsal root ganglion (DRG) neurons demonstrated that Charcot-Marie-Tooth disease (CMT)-associated mutations of the low-molecular-weight neurofilament protein (NF-L) decreased mitochondrial lengths and disrupted mitochondrial fusion and movement in axons [97].

The majority of axonal neurofilament subunits are synthesized in the soma and subsequently transported into axons along microtubules [98]. Accumulation of neurofilaments in the cell bodies and proximal axons, due to an imbalanced expression of neurofilament subunits, altered PTMs of neurofilament proteins, or impaired axon trafficking has been identified as a common feature in multiple neurological disorders, including CMT, ALS, PD and AD [99,100]. There is evidence that mRNAs of neurofilament proteins reside in axons [101,102] and are also locally translated there [103,104]. However, the functions of these locally synthesized proteins are yet to be discovered.

Dynamic and diverse axonal actin structures play important roles throughout development and adulthood, from axon specification, initiation, elongation, guidance, branching to the development of presynaptic terminals [105]. In developing axons, actin filaments are enriched in the peripheral region of growth cones, where they form dynamic lamellipodia and filopodia to facilitate axonal pathfinding [106]. Upon target arrival, actin polymerization is also required for axon arborization [107]. As first observed by super-resolution microscopy, actin is organized in ring structures underneath the plasma membrane in mature axons, which are connected and evenly spaced by spectrin heterotetramers [108]. Such actin ring-spectrin structures together with other interacting proteins form membrane-associated periodic skeletons to support axon architecture by conferring elasticity and stiffness [109]. At the presynapse, actin filaments accumulate at the active zone and associate with synaptic vesicles to promote active zone formation and to regulate synaptic vesicle clustering [110,111]. Conceivably, dysregulation of actin localization and organization can exert a detrimental effect on axon development and survival. Missense mutations in one of the two neuronal actin isoforms, β-actin and γ-actin, have been reported in neurological diseases, including juvenile-onset dystonia [112], late-onset sensory-neural deafness [113] and Baraitser–Winter syndrome [114].

It has been well established that locally synthesized β-actin proteins function in axon steering and branching in developing neurons [48,49,115,116], but the extent of their involvement in mature axons and disease-affected neurons remains to be explored. Early studies demonstrated that whilst *β-actin* mRNA localizes to axons, *γ-actin* mRNA is restricted to the soma in developing cortical and adult DRG neurons in cultures [104,117]. However, a recent piece of work challenged this view by showing the localization of *γ-actin* mRNA in developing cultured motor axons using qRT-PCR and fluorescence in situ hybridization [118]. In the same study, local translation of *γ-actin* mRNA in growth cones and branch points was also demonstrated by a FRAP assay using reporter constructs [118], suggesting that axonally synthesized actin isoforms may differ between different types of neurons. In addition to actin proteins, actin-associated proteins, such as α-spectrin, were identified in an axonal translatome of mouse retinal neurons [9], suggesting LPS could be involved in the dynamic regulation of axonal actin organization. This could help to provide structural stability and plasticity during axon development and maintenance.

### Microtubule-based transport is critical to axonal trafficking

The microtubule cytoskeleton is critical for long-range transport in axons, and therefore for LPS. In this transport system, anterogradely and retrogradely transported cargos, including mRNAs and translational machinery components, are loaded onto motor proteins, which move along polarized microtubule tracks. Conventionally, axonal trafficking is considered to feature two distinct transport modes, namely fast and slow [119]. Fast axonal transport (0.5–5 µm/s) mainly carries organelles and ribonucleoprotein (RNP) granules [79], including complexes carrying disease-related proteins (e.g. APP, Huntingtin) [120,121], whilst slow axonal transport (0.01–0.001 µm/s) carries cytoskeletal components, such as neurofilament proteins [122]. Both modes of axonal transport are carried out by the same microtubule-based motor proteins, anterogradely-moving kinesins and retrogradely-moving dynein. The difference in their average velocity results from the occurrence of prolonged pauses in movement during slow axonal transport [123], which is modulated by dynamic attachment of multiple motors to the cargo [124]. Increasing evidence suggests that fast axonal transport defects are more common in neurological disease-affected neurons, possibly as a result of mutations in proteins mediating fast axonal transport, or trafficking perturbation in cargos undergoing fast axonal transport [125]. Besides determining the speed, cargo attachment to opposing motors allows them to undergo bidirectional transport and frequently change direction, which requires coordination of motor activities, including the duration of individual motor attachment and run lengths in either direction [126,127]. Given the role of axonal transport in delivering structural components, organelles and survival signals, it is not surprising that mutations in motor proteins and their cofactors cause a wide range of neuropathies [128].

Mutations and aberrant post-translational modifications in tubulins lead to multiple neurodevelopmental and neurodegenerative diseases, including ASD, polymicrogyria, ALS, and AD [129,130,131], which could potentially be partly due to errors in local synthesis of these proteins. mRNAs encoding tubulins have been detected in axons in several transcriptomic studies (Fig. 1) [10,40,41]. Moreover, radioactive labelling and proteomic studies have identified several locally synthesized tubulin proteins [34,132,133]. Although these form <1% of the total axonal β-tubulin pool, according to [^35^S]-Met radioactive capturing analysis [132], this does not disprove the importance of axon-derived tubulins [131], as different tubulin isoforms [134] or PTMs [135] may be enriched in the somatically and axonally synthesized pools, resulting in distinct functionalities. Inhibiting the local synthesis of β2B-tubulin, which mainly localized to the growth cone periphery, resulted in growth cone collapse in cultured DRG neurons [136]. Mutations in β2B-tubulin gene were found in patients diagnosed with polymicrogyria [137,138], but the extent to which axonally expressed β2B-tubulin contributes to the disease needs further research.

Microtubule-associated proteins actively regulate the stability and dynamics of microtubules in axons, and their functional impairments often lead to axonopathy. One of the most extensively studied axonal microtubule-associated proteins is tau, which is important for microtubule stability and implicated in disease [139]. A range of neurological disorders (termed ‘tauopathies’) is characterized by deposition of hyperphosphorylated tau protein in the brain, including AD and frontotemporal dementia (FTD). In axons, tau is reported to facilitate the organization of distal microtubules, which is important for axon trafficking, outgrowth and navigation [140,141]. *tau* mRNA contains an axonal localization signal and is locally translated [142,143], but the phosphorylation level of axonally synthesized tau is yet to be determined. Intriguingly, functional and pathogenic heterogeneity exists between the six tau splicing isoforms [144,145]. Therefore, characterization of the isoform-specific role of axon-derived tau would provide insights into its functional significance, which is particularly relevant in disease models. In mature healthy neurons, tau proteins are almost exclusively localized to axons, but somatodendritic tau inclusions are frequently found in AD-affected neurons [146]. It is worth noting that, although localized tau synthesis is restricted to axonal compartments, *tau* mRNA is also localized to dendritic spines. Activation of glutamate receptors triggers local synthesis and hyperphosphorylation of tau in dendrites, leading to somatodendritic accumulation of hyperphosphorylated tau [147]. This has been shown to be a key step in the initiation of tauopathies [148], indicating the importance of correct *tau* mRNA localization. Besides tau, another axonally synthesized microtubule-associated protein ‘mitogen-activated protein kinase kinase 7ʹ (MKK7) has also been shown to promote microtubule bundling and neurite elongation by correctly positioning Jun ‘N-terminal kinase’ (JNK) signalling in axon shafts [149].

There is also some evidence that LPS of motor proteins contributes to or regulates axonal transport, which further establishes a link between the two processes. Detection of *kinesin* mRNAs in giant squid axons and dynein light chain mRNAs in rodent axons have been reported over two decades ago [150; 151] and recent axon-TRAP and proteomics-based translatomic studies subsequently revealed that many of the motor protein mRNAs are actively translated, including kinesin-1 proteins (KIF5A, 5B and 5 C) and a kinesin-3 protein KIF1A [9,39,152]. Of these, KIF5A localizes predominantly to axons rather than dendrites in cultured hippocampal cells [153], and KIF1A is a major axonal motor responsible for long-distance transport of synaptic vesicle precursors and neurotrophin-containing dense core vesicles [154,155]. Mutations in or hyperactivation of KIF1A are associated with neurodegenerative disorders, such as hereditary sensory and autonomic Neuropathy Type 2 and hereditary spastic paraplegia [156,157,158]. It will be of interest to determine the role of axonally synthesized kinesins and their link to kinesin-related diseases. In addition, local on-demand production of dynein cofactors has been demonstrated to mediate retrograde transport in healthy and disease-affected axons. Two dynein cofactors are differentially translated upon nerve growth factor (NGF) stimulation or withdrawal in axonal compartments: Lis1, a force-generating component in the dynein complex, and p150^Glued^, one of the eleven subunits of dynactin. Therefore, a local translation-based mechanism to regulate stimulus-specific retrograde trafficking has been put forward [159].

### Neuropathy-related RNP condensation regulates axonal mRNA transport and localization

The mechanism of axonal mRNA localization to support LPS is evolutionally conserved in different cells and organisms: loading of mRNAs onto motor proteins is facilitated by RBPs that recognize localization elements often present at the 3ʹUTR [160,161,162]. Structurally, a majority of RBPs consist of RNA-recognition motifs (RRMs) and intrinsically disordered domains (IDDs), the latter being regions with low sequence complexity and no fixed three-dimensional structure. Gene ontology annotations reveal that a third of human IDD-containing proteins function in RNA-binding [163], illustrating heavy involvement of IDDs in RBP functionalities. IDDs together with RRMs allow RBPs to flexibly and multivalently interact with multiple protein/RNA targets to reversibly form membraneless organelles or granules (a liquid-liquid phase separation, LLPS). This can locally concentrate granule constituents and hence promote physical interactions between these molecules [164]. The strength of their interactions is sensitive to temperature, pH and salt concentration [165], and can be further fine-tuned by various protein PTMs [166], providing additional layers of regulation. However, these useful and unique properties of RBPs are the same feature responsible for their role in the development of neurodegenerative disease. Indeed, structural and functional alterations of a subset of RBPs are over-represented in patients diagnosed with ALS, FTD and AD [167]. When intracellular phase transitions become dysregulated, resulting in hyper-stable RNP granules, proteins and RNA could become irreversibly trapped within the granules, preventing them from performing normal functions, including LPS. Despite being regarded as pathological hallmarks in neurodegenerative diseases, it is under debate whether RNP depositions on their own are pathogenic. It has been proposed that they instead serve as a reporter for the pathogenic dysregulation of cellular processes that often precedes aggregate formation [168]. Therefore, rather than focusing on approaches to ‘dissolve’ these aggregates, it may be more relevant to identify the dysregulated processes that promote hyperstable RNP granule formation.

Previous studies have demonstrated that RBP phase transitions are sensitive to and partly regulated by local protein concentration, RNA concentration and conformation, PTMs, and the availability of chaperones and other binding partners [169]. Consequently, aberrant homoeostasis of any of these factors may enhance the tendency for pathological aggregates to form and persist during disease progression. For instance, RBP:RNA ratio, RNA lengths and secondary structures, and their RBP binding specificity jointly determine the predominant material states and dynamics of RNP granules [170]. As a result, the presence of sub-optimal amounts and species of axonal RNAs may reduce axonal trafficking, exacerbating the disruption of local homeostasis in diseased axons in a negative feedback loop. In addition, the link between aberrant RBP PTMs and neurological disorders has also been recently established. PTMs can effectively alter the strength of intra- and intermolecular interactions by modifying electrostatic charges of amino acids, hydrophobicity and protein structures, for instance, serine/threonine/tyrosine phosphorylation, arginine methylation, and arginine citrullination. Therefore, PTMs are powerful modulators of RBP LLPS and dynamic RNP granule regulation [166], which can be deregulated in disease. For instance, FUS inclusions with unmethylated arginine have been found in FTD patient post-mortem tissue [171,172]. Arginine hypomethylation promotes the formation of cytoplasmic FUS inclusions, and axons expressing hypomethylated FUS showed an increased number of axonal FUS-containing granules accompanied by compromised LPS [58]. This study also showed that the reduced LPS could be effectively restored upon overexpression of a FUS chaperone, Transportin-1, which imports FUS from the cytoplasm into the nucleus and represses FUS aggregate formation [173,174,175]. Changes of LPS in response to FUS hypomethylation and the level of its phase modulator support a close link between PTMs, chaperones, phase separation and LPS in axons.

The neuronal context of spatially confined axonal compartments packed with high density of cytoskeleton and organelles and unique modes of RBP transport may further enhance pathological RNP assembly. Under these conditions, protein and RNA may be concentrated locally, elevating local axoplasmic viscosity and influencing RBP phase behaviour [84]. This can occur in several ways: 1) a regional disruption of axonal transport in response to local stimuli or insults; 2) a burst of LPS, especially of IDD-containing RBPs identified as highly locally translated in axonal translatomic studies, including FUS and hnRNPs [39]; 3) active recruitment of proteins and RNAs by membrane-bound organelles. Recent evidence showed that a proportion of RNP granules ‘hitchhike’ on membrane-bound organelles, such as peroxisomes, mitochondria and endosomes, acting as vehicles for RNP granule trafficking and localization [176,177,178], in contrast to the conventional view that RNP granules undergo long-range trafficking through direct tethering to motor proteins. In vertebrate axons, late endosomes act as platforms to recruit mRNAs and translation machinery to support LPS [179]. Disruption of this process can be disease-causative: CMT2B-associated mutations of Rab7a attenuate LPS in axons, compromise mitochondrial function and eventually result in axon degeneration. In addition, ALS-associated mutations of an adaptor between lysosomes and RNP granules, annexin A11, impair its intra-axonal phase-transitioning ability and its tethering between RNP granules and lysosomes, resulting in perturbed RNA localization in axons [180].

These observations open up an exciting direction for future research into how axons organize local translation into micro-domains and regulate translation specificity in these sub-compartments. As a main driving force for RNP granule formation, LLPS may also contribute to the establishment and stabilization of organelle-RNP compartments, as demonstrated by annexin A11 tethered to lysosomes [180]. The role of such molecular anchors remains to be explored for other organelles. Furthermore, it has been reported that translation only takes place on the surface of late endosomes in *Xenopus* RGC axons, although both early and late endosomes associate with key components of translational machinery, including mRNA, RBPs and ribosomes [179]. This leads to the question of what activates translation on these RNP-bound organelle platforms. The physical location of the organelles may be a key factor: organelles and RNPs are highly enriched at branch points and axon terminals, where high levels of translation activity often occur [116,181]. It is possible that the local density of organelles and recruited molecules concentrates components required by translation or alters the physical states of the surrounding micro-environment to promote translation. Alternatively, translation activity could be modulated by certain regulatory elements associated with individual organelles, such as miRNAs [182]. Another open question lies in the control of mRNA localization and translation specificity on platforms; recruiting specific RBPs and the subset of mRNAs bound to them could be a way to define the identity of a translation hub. Finally, whether the disruption of micro-domain arrangement and regulation is prevalent in neurological disease-affected neurons remains to be investigated.

## Establishment of axon architecture and connectivity

In order for appropriate connectivity between neurons and target cells to be generated and maintained, axonal branches and even whole neurons are at times remodelled. To establish and specify their innervation fields, developing axons from terminal branches with diverse lengths, density and complexity, allowing them to synapse with multiple target cells simultaneously, with excess synapses being pruned at later stages [183]. Local translation is known to have a role in branching of axons. Data from chick embryonic sensory neurons suggest that NGF promotes axon branching by modulating the actin cytoskeleton, in part via stimulation of LPS through phosphoinositide 3 kinase (PI3K) signalling [181]. Furthermore, RNA granules dock at the bases of new branches and invade stable branches, and local synthesis of β-actin at these sites is important for axon arbour dynamics [116]. There is also some preliminary evidence that presynaptic LPS is important in the pruning stage of development, which can intersect with its role in survival signalling. For example, in degeneration-like pruning in the PNS, competition for neurotrophic support is an important driving force [184], and neurotrophin-stimulated LPS is important for this response [185].

In neurological disorders, branching and/or pruning are often compromised. This is perhaps intuitive for neurodevelopmental disorders such as for FXS, but more recent findings imply axonal structure may also be affected in neurodegenerative diseases. The association of these defects with RBPs has been demonstrated for several such disorders, which can to some extent be linked to LPS.

### RBP dysregulation compromises axon branching and pruning in neurodevelopmental disorders

In FXS, a clear link between RBP dysregulation and compromised neuronal connectivity exists, which makes it an important case study. We briefly discuss this link, and then outline the evidence that FMRP affects presynaptic translation of proteins important for axonal structure and function. We then indicate the extent to which similar processes are implied in other neurodevelopmental disorders, namely ASD and epilepsy.

In FXS, loss of function of the RBP FMRP results in defects in synaptic formation and plasticity. It is well-known that dendritic spine structure is altered in FXS, with more but longer, potentially immature spines being observed [186]. *dfmr (fmrp1* homologue) knockout in *Drosophila* results in axonal overgrowth and overbranching, which compromises synapse formation [187]. However, decreased connectivity at certain developmental stages has also been reported in FXS models, along with more ‘diffuse’ axon arbours, with a higher connection density along the barrel borders and reduced connectivity at the centre [188]. This is consistent with a pruning defect [186].

Some of the effects of loss of FMRP function are likely due to regulation of LPS being compromised: FMRP is known to be a negative regulator of translation [189], and several observations suggest it locally regulates translation at synapses [190]. Consistent with it having a functionally important role in regulating LPS, FMRP associates with polyribosomes and disruption of this interaction causes particularly severe disease, via the rare I304N mutation in the ribosome-interacting KH-domain [191]. FMRP-mediated regulation of LPS is known to be important in dendrites, where it influences activity-dependent long-term potentiation. For instance, an imaging study showed knockout of *fmr1* prevents an increase in levels of the presynaptic protein CamKIIα upon group I metabotropic glutamate receptor stimulation, which was demonstrated to be protein synthesis-dependent by cycloheximide treatment and presumed to be local due to its ten-minute timescale [192]. However, FMRP is increasingly recognized to be important for regulation of presynaptic translation as well [193]. In particular, FMRP-containing granules are found in a subset of axons, most prominently during synapse formation and pruning [194,195], indicating a possible presynaptic role of FMRP in synapse formation [196]. Notably, this association is not limited to early developmental stages: FMRP-containing granules are also found in a subset of mature mammalian axons (but not dendrites), where they associate with ribosomes as well as (a subset of) FMRP mRNA targets [197].

Several key axonal mRNA targets of FMRP have now been identified, which have a range of functions during different developmental stages. In hippocampal neurons, FMRP has been shown to be involved in the LPS-based response to the guidance cue Sema3A during axon extension, including by promoting the local synthesis of the microtubule-associated protein 1B (MAP1B) [198]. Previously, it had been shown that double knock-out of *dfmr* and *futsch* (the *Drosophila map1b* homologue) could rescue synaptic structural defects in the eye and neuromuscular junction [199]. During presynapse formation in mouse cortical neurons, FMRP negatively regulates local translation of the synaptic vesicle fusion protein Munc18-1, as demonstrated in cultured mouse cortical neuron axons that were physically separated from the soma [200]. In *Drosophila*, it has been shown that FMRP functions in axon maturation in two distinct ways: it inhibits axon growth during late pupal development, and functions in activity-dependent pruning in emerging adult flies, during which time its activity correlates inversely with levels of the profilin homologue chickadee [201]. Though this link has not been demonstrated to be due to regulation of LPS of chickadee (an actin-remodelling protein), *chickadee* mRNA has been shown to localize to remodelling *Drosophila* axons, with its mislocalization resulting in remodelling defects [202].

There are implications that perturbed phase separation of FMRP can occur in FXS, though the link to dysregulated LPS is not yet firmly established. Notably, it has recently been found that only certain splicing isoforms of FMRP reduce axonal arbour complexity when overexpressed [203]. This regulation of arbour complexity does not seem to require the RNA-binding domains, including the KH-domain, but does require an intact nuclear export signal as well as the presence of a phosphorylatable serine that regulates translational suppression in FMRP-associated polyribosomes [203,204]. Instead, the I304N (KH-domain) mutant was found to be more prone to fibril formation, indicating that this mutation may affect translation by deregulating FMRP granule phase state rather than simple loss of function of RNA or ribosome binding [203]. In support of this theory of perturbed FMRP phase behaviour in certain disease variants, rare FXS-associated mutations in the *fmr1* coding region cause loss of cytoplasmic FMRP1 function through introduction of a nuclear localization signal [205]. This induces nucleolar aggregation of FMRP1 [205], which is consistent with a phase separation behaviour (where increased local concentration makes phase separation and subsequent aggregation more likely). As FMRP has recently been demonstrated to phase separate, which was suggested to be important for activity-dependent translation regulation [206], this raises the interesting idea that perturbation of its phase behaviour may be harmful to local proteomic homoeostasis. Its aggregation would result in cytoplasmic loss of function of FMRP-associated mRNAs, and so could putatively have the same functional consequences as mutations causing nonsense-mediated decay of its *frmp* mRNA.

There is also evidence that dysregulated RBP activity occurs in other neurodevelopmental disorders that feature altered synaptic connectivity, such as ASD and epilepsy, but the links to altered connectivity and LPS have not been directly established for most of these RBPs. Notably, FXS is comorbid with select variants of these diseases [207]. Epilepsy can arise through acquired brain lesions, but also during the development of the cortex, at the steps of neuronal proliferation, neuronal migration, or synaptic refinement [208]. For instance, tissues from patients with mesial temporal lobe epilepsy recurrently display the aberrant formation of excitatory connections due to sprouting of hippocampal dentate granule cell axons into the dentate inner molecular layer [209]. Deficiencies in several RBPs other than FMRP have been associated with epilepsy, including BRUNOL4/CELF4 [210], RBFOX1 [211], and Pumilio2 [212]. Of these, Pumilio2 is suggested to affect LPS: it is present in dendritic stress granules during metabolic stress [213] and has recently also been reported to influence the transcriptome of the developing axon by somatic retention of certain mRNAs [214]. Other RBPs implicated in epilepsy are known to be regulated by the translation initiation-promoting mTOR/MAPK pathway, pharmacological inhibition of which effectively prevents epileptogenesis [215]. Axon pathology is thought to be at the core of aberrant connectivity in ASD, with changes in axon diameter, myelination and branching being observed in a range of studies [216]. Multiple ASD-associated genetic alterations have been identified as contributing to some of these changes in axon architecture, such as in the gene encoding chromatin remodelling protein ‘chromodomain helicase DNA-binding protein 8ʹ (CDH8) [217] and in the *ANK2* gene, which encodes two major ankyrin polypeptides that are important for polarized transport of organelles [218]. However, ASD is also linked to deficiencies in several RBPs, including RBFOX1 [219], CSDE1 [220], and Caprin1 [221]. For CSDE1, a link between its function and aberrant connectivity has been established, though the functional importance of LPS remains to be investigated: knockdown in primary mouse cortical neurons leads to an overgrowth of the neurites and abnormal dendritic spine morphology/synapse formation [220].

### RBP variants associated with neurodegenerative diseases also affect axon architecture

Several mutations in RBPs associated with neurodegenerative diseases, with different ages of onset, have also been shown to affect axonal architecture. Here, we review the evidence linking the RBPs SMN, TDP-43, and FUS to axonal structural defects, and consider to what extent these links might be attributable to dysregulation of LPS.

SMN is a ubiquitously expressed RBP, reduction in the levels of which results in selective dysfunction of motor neurons (spinal muscular atrophy; SMA) [222]. SMN localizes to branch points and growth cones in the axons of primary cultured motor neurons [223], and its depletion has been shown to affect motor neuron axon architecture in several model systems. In zebrafish embryos, knockdown of SMN causes defects in motor neuron axonal outgrowth and pathfinding in a cell-autonomous manner, a phenotype that is not seen in other neuronal subtypes [224]. Using a mouse model of SMA, it has been shown that the earliest structural defects occurred at the neuromuscular junction, and included poor terminal arborization and formation of intermediate filament aggregates [225]. In another mouse model of SMA, it has been demonstrated that reduction of SMN levels also results in abnormal synaptogenesis and neurofilament accumulation in retinal neurons [226]. This study also suggested that SMN-deficient retinal neurons displayed a defect in axon outgrowth, as a reduced number of axons in the optic nerve were observed without a decrease in the number of retinal ganglion cells [226].

Several studies indicate that SMN affects LPS of proteins important for the correct establishment of axonal architecture and connectivity. SMN interacts with the RBP HuD [227], with which it is co-transported in axons of mouse primary motor neurons, and knockdown of SMN reduced both axonal HuD and axonal poly(A) mRNA levels, indicating that it has a role in facilitating axonal localization of certain mRNAs [228]. In particular, reduction of SMN levels is associated with reduced axon outgrowth of motor neurons, which correlates with reduced axonal levels of *β-actin* mRNA, the 3ʹ-UTR of which is bound by SMN’s binding partner hnRNP-R [229]. In the motor neurons of developing zebrafish embryos, hnRNP-R knockdown resulted in reduced axonal outgrowth associated with loss of *β-actin* mRNA in the growth cone, without motor neuron death or defects in dendrite outgrowth [230]. SMN not only affects LPS by influencing mRNA localization, but also affects LPS rates directly. In particular, it has been demonstrated to regulate axonal translation via the miRNA miR-183: in SMN-deficient neurons, miR-183 levels are increased, which results in reduced local translation of the protein mTOR, a key stimulator of LPS [16]. Furthermore, it has now been shown that SMN deficiency severely disrupts LPS within motor neuron axons and growth cones, and that rescue of localization of the SMN target mRNA encoding ‘cytoskeleton-associated growth-associated protein 43ʹ (GAP43) can rescue axon outgrowth defects in SMA neurons [231].

The ALS-associated protein TDP-43 is increasingly recognized to affect motor neuron axon structure, which may be due to its regulation of axonal mRNA localization. Expression of ALS-associated human variants of TDP-43 in zebrafish embryos caused motor neuron defects, with shorter axons and premature and excessive branching being observed [232]. This effect was phenocopied by knockout of the zebrafish homologue of TDP-43, indicating a loss-of-function mechanism, though a neurotoxic gain-of-function effect associated with TDP-43 mutant aggregation was observed in dissociated spinal cord cultures [232]. It has been suggested that TDP-43 regulates axonal outgrowth in motor neurons by post-transcriptional regulation of cytoplasmic mRNAs, since it was found to be actively transported into axons of primary cultured motor neurons, where it colocalizes with known axonal RBPs [233]. Like for FMRP, loss of function of TDP-43 affects cytoskeletal architecture: knockout affects synaptic growth and bouton shape at the *Drosophila* neuromuscular junction [234,235], which is associated with reduced levels of Futsch (the *Drosophila* MAP1B homologue) in distal axons, the mRNA of which is bound by TDP-43 [234]. The structure of the *Drosophila* mushroom body was similarly affected by overexpression of TDP-43, with smaller axonal lobes being observed [235]. Therefore, it may similarly be speculated that disease-associated variants of TDP-43 affect axonal function through structural alterations associated with changes in LPS of cytoskeletal and/or cytoskeleton-associated proteins.

There is also evidence that ALS-associated mutations in FUS affect axon branching, though the nature of the effect may depend on the neuronal subtype and mutant variant studied. In cultured primary cortical cells, expression of FUS-R521C led to a reduction in the number of primary axonal branches, when compared with wild-type neurons or neurons expressing wild-type FUS [236]. These defects were linked to the interaction of FUS with SMN: mutant FUS interacted more strongly with SMN and perturbed its axonal localization, and overexpression of SMN was able to rescue the branching defects induced by mutant FUS [236]. In human-induced pluripotent stem cells differentiated into motor neurons, mutant variants of FUS (patient-derived or genome-edited) resulted in increased axonal branching [237]. This effect was rescued by suppression of aberrant expression of transcription factor FOS-B, the mRNA of which was detected in axon bundles and is bound by FUS, and which was also found to be abnormally upregulated in ventral horn neurons in autopsy samples of ALS patients [237]. Together with the observation that endogenously expressed FUS is known to affect LPS in axonal growth cones of *Xenopus* retinal ganglion cells [58], this suggests regulation of LPS by FUS might occur in axons, which could play a role in determining axon architecture.

## Axonal survival signalling

**Figure 4.**
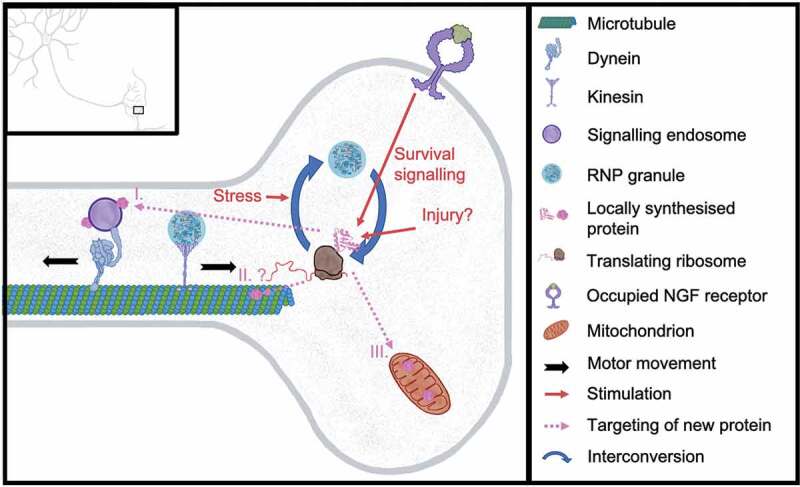
**Selected contributions by LPS to synaptic survival and adaptability**. LPS in the presynaptic terminal contributes to a range of processes important for neuronal maintenance, including I. survival signalling, II. remodelling of cytoskeletal elements, and III. maintenance of mitochondria

After axons establish their innervation fields through branching, pruning and presynapse formation, intricate crosstalk between signalling pathways and metabolic processes involving pro-survival factors and organelles comes into play to support the health and survival of mature axons. Early research proposed axon degeneration occurs as a consequence of cell body death, due to insufficient protein and energy support from the soma [238]. This view was first challenged by the identification of the Wallerian degeneration slow (Wld^S^) protein, which delays degeneration of somaless axons for weeks [239]. Wld^S^ was subsequently shown to substitute for activity of the labile protein nicotinamide mononucleotide adenylyltransferase 2 (NMNAT2), an axon survival factor with both foldase and NAD^+^ synthase activity [240]. However, it has since been demonstrated that NMNAT2 depletion upon axotomy activates a specific axonal degeneration programme via the downstream effector SARM1 [241], and that modulation of this downstream effector’s activity rather than NMNAT2 activity can rescue the lethality of NMNAT2 deprivation [242], indicating axon degeneration upon injury is initiated by specific signalling pathways. Indeed, more evidence has now accumulated that demonstrates that axons rely on multiple axon-initiated pathways for survival [14,15,185] (Fig. 4).

The most well-established mechanism to promote axon survival relies on the binding of target-derived neurotrophic factors secreted by target cells, including NGF, BDNF, neurotrophin 3 and 4 (NT3 and NT4), to their receptors TrkA, TrkB, TrkC and p75 on axonal membranes [243]. Upon binding to neurotrophins, receptors are internalized, forming signalling endosomes, and subsequently retrogradely transported to the soma by dynein motors [244], where they activate trophic signalling pathways, including PI3K and mitogen-activated protein (MAP) kinase cascades [245,246,247]. This leads to changes in transcriptional profiles of the stimulated neurons through induction of various transcription factors, including cyclic AMP responsive element-binding protein (CREB), which promotes neuronal survival [248,249].

Pruning and apoptosis are respectively triggered by local or global loss of survival signalling via NGF and the TrkA receptor [250], which has downstream effects on both anti-apoptotic signalling and the NMNAT2/SARM1 pathway [238]. Interestingly, several components of these pathways act at least in part on the mitochondria. The anti-apoptotic protein Blcw is found in axons [251], which is part of the Blc-2 family of proteins that represses the mitochondrial permeability transition that is key in apoptotic signalling [252], and its loss in small fibre sensory neurons is associated with mitochondrial abnormalities and primary axonopathy [251]. Furthermore, Wld^S^ increases basal mitochondrial mobility and calcium buffering [253]. Therefore, these organelles are a signalling hub in survival signalling, in addition to being important for LPS. Here, we discuss the various intersections between axonal survival signalling, LPS, and mitochondrial function.

### Axonal LPS transfers information in survival signalling

The contribution of LPS to soma-independent axonal survival pathways first came to light with the discovery that axonally synthesized Lamin B2 (LB2), an intermediate filament protein, is critical in preventing axonal degeneration but not in axon guidance, which was made using the model system of developing *Xenopus* RGC neurons [14]. Proteomic screening demonstrated that stimulation with the guidance cue engrailed-1 affected LPS of several hundred proteins, with the most robust increase in axonal synthesis rate occurring for LB2. The localization of *lb2* mRNA and its local translation were then respectively confirmed by fluorescence *in situ* hybridization and by quantitative immunofluorescence in the presence and absence of translation inhibitor anisomycin. To further validate that *laminb2* mRNAs are translated in RGC axons *in vivo*, a grafting experiment was combined with an axon-TRAP assay. First, eye primordia from a donor embryo expressing GFP-tagged ribosomal protein L10a were transplanted to a host wild-type embryo. After exiting the eye, GFP-RPL10a-positive RGC axons innervated the contralateral wild-type brain hemisphere. Next, pulldown of ribosome-bound mRNAs from the host brain lysates, using the GFP-RPL10 as a ribosome tag localizing exclusively to RGC axons, confirmed LB2 was indeed associated with ribosomes in RGC axons. It was then demonstrated that axonally synthesized LB2 is important for axonal survival: electroporation of a translation-blocking antisense morpholino for *laminb2* mRNA into distal axons *in vivo* resulted in axonal death without cell body death after extension into the optic tectum, without retrograde transport of the morpholino being detectable, and expression of exogenous LB2 lacking a nuclear localization signal could almost completely rescue the degenerative phenotype.

LPS of survival-related proteins is now also known to be triggered by neurotrophin signalling. Neurotrophin signalling-related mRNAs have been identified in a range of axons (Fig. 1) [9,39,41]. For instance, NGF derived from target cells is detected by sensory axons during development, stimulating axonal translation of CREB, which is retrogradely trafficked and promotes neuronal survival [254] (Fig. 4). Furthermore, neurotrophins can promote axon survival by stimulating local translation of anti-apoptotic proteins [185]: using compartmentalized cultures of dorsal root ganglion cells stimulated with NGF and BDNF, it was demonstrated in that *blcw* mRNA is transcribed in response to retrogradely transported neurotrophins, which is then transported to axons and translated into the anti-apoptotic protein Bclw. Neurotrophins may also regulate the local translation of *blcw* mRNA, in addition to its transcription and transport: cycloheximide addition to the axonal compartment prevented the increase in axonal Blcw observed upon extended neurotrophin stimulation, whilst addition to the somal compartment had no such effect. Importantly, inhibition of local translation prevented neurotrophins’ survival-promoting effects, and was associated with increased activity of caspase 6, which is inhibited by Blcw. Protein transfection of Blcw into axons protected from neurotrophin withdrawal-induced axonal degeneration, further indicating LPS of this protein is particularly key in axonal survival.

Disruption of LPS has, to our knowledge, not yet been shown to be causative in specific diseases associated with disrupted survival signalling. However, it is known that local loss of survival factors can contribute to disease. In TDP-43-associated ALS, for example, there is splicing defect-associated loss of the survival factor stathmin-2 (STMN2), a microtubule-destabilizing factor essential for axonal microtubule integrity, resulting in impairment of neurite growth and neuronal repair after injury [255]. Restoring levels of this survival factor could rescue TDP-43-associated phenotypes in human pluripotent stem cell-derived human motor neurons [255]. Notably, it has been suggested that STMN2 (also known as superior cervical ganglion 10, SCG10) is locally synthesized in response to axonal injury in proximal axons [256] (Fig. 4), and it is prominent in a range of axonal transcriptomes (Fig. 1) [9,10,40]. Furthermore, in a mouse model of SMA, it has been shown that mutation of SMN causes a reduction of muscle cell secretion of C1q/TNF-Related Protein 3 (CTRP3), which in turn regulates axonal LPS via the mTOR pathway, including SMN itself [257].

### Axonal mitochondria are closely associated with LPS and axon survival

As uncovered by a series of studies examining local components essential to axon viability, axonal mitochondria have been increasingly recognized to contribute to axonal integrity and survival. Suboptimal mitochondrial activities, which fail to provide sufficient energy, metabolites and calcium buffering, may result in comprised axon survival [258]. Experimentally, it has been demonstrated that the presence of mitochondria in axons of *C. elegans* protects against degeneration following axotomy [259]. In fact, mitochondrial dysfunctions are known to be associated with several neurodegenerative disorders with prominent axonal phenotypes [260,261], suggesting that axons are particularly sensitive to disturbance to mitochondrial integrity. For instance, mutations of mitochondrial proteins and lamins may cause Charcot-Marie-Tooth type 2B (CMT2B) diseases, an inherited neuropathy characterized by sensory axon degeneration [262,263]. Similarly, CMT2A is commonly caused by mutations in the gene encoding the mitochondrial protein mitofusin-2 (MFN2) and is associated with degenerative changes in axonal mitochondria in patient sural nerve biopsies [264]. MFN2 promotes inter-mitochondrial fusion as well as tethering of ER to mitochondria; compromising of this latter function (rather than altered bioenergetics) may be the main cause of pathologically altered mitochondrial morphology and transport in CMT2A, as has recently been reported in patient-derived fibroblasts as well as mutation-carrying primary mouse motor neurons [265,266].

Mitochondrial function is linked to LPS as well as to axon survival, since mitochondria likely play an active role in LPS as a local energy source [267]. Their localization is affected by local energy demands: globally, signalling energy consumption of neurons and their subcellular compartments correlates with mitochondrial positioning, with dendrites using over half of the energy required for signalling, and containing over half of the mitochondria [25,268]. Furthermore, mitochondria cluster to locations with high rates of LPS: dendritic mitochondria are stably ‘compartmentalized’ to provide ATP for activity-dependent LPS, with mitochondrial filaments of around 30 μm being anchored near spines by tethering to the cytoskeleton [269]; in axons, mitochondria accumulate at branch points, which contributes to actin-dependent branching [116,181].

Importantly, one of the major categories of mRNAs that is localized to and translated in axons *in vivo* is those related to mitochondrial function [9] (Fig. 1), suggesting that axon-resident mitochondria require a local supply of proteins for their upkeep. A recent publication suggests LPS is important for mitochondrial maintenance at synapses: stimulation of synaptosomes with NMDA and glutamate induced LPS of mitochondrial proteins, which were shown to be incorporated into respiratory complexes by radiolabel tracing, and perturbation of LPS by knockout of *fmr1* was associated with morphology defects in synaptosome mitochondria [270]. Therefore, axonal mitochondria potentially both maintain and are maintained by LPS, making LPS of mitochondrial proteins key for continued axon survival: disruption of mitochondrial function may compromise LPS, which then, in turn, compromises mitochondrial function, and vice versa.

Loss of mitochondrial function triggers degenerative pathways, including following compromised LPS of key mitochondrial proteins. Depolarization of the mitochondrial membrane activates the Wallerian degeneration pathway [271], and is a key step in the apoptotic pathway generally as part of the mitochondrial permeability transition [272]. As shown in multiple studies, loss of maintenance of axonal mitochondrial membrane potential is associated with compromised axonal integrity [14,179,273,274]. This can arise as a consequence of attenuation of local mitochondrial protein production, as was demonstrated for LB2: axonal LB2 localizes to mitochondria, and local depletion of LB2 results in a significantly reduced mitochondrial membrane potential and elongated morphology, which is indicative of mitochondrial dysfunction [14] (Fig. 4). Inhibition of LB2 local translation caused axon degeneration by disrupting mitochondrial function and altering mitochondrial trafficking in axons. As phosphorylation of LB2 triggers nuclear membrane fragmentation during cell division [262], LB2 might control mitochondrial membrane cleavage during mitochondrial fission, which could explain the observed elongated mitochondrial morphology and decreased membrane potential in LB2 knockdown axons. *laminb2* mRNA is transported into axons by the RNA-binding protein SPFQ [14,275], rare fALS-associated variants of which mislocalize away from axons [276], and on late endosomes [179]. These endosomes localize to the proximity of mitochondria, and are known to act as translation platforms for local synthesis of mitochondrial proteins, a process that is perturbed by mutations associated with Charcot-Marie-Tooth type 2B neuropathy [179].

## Neuronal stresses and stress responses

Given their long lengths and large surface areas, neurons are likely to be exposed to environmental insults that, if not dealt with, may perturb intracellular homoeostasis, resulting in impaired neuronal functions and potentially jeopardizing their long-term survival. Some of these insults are unique to the nervous system, such as compartmentalized stresses, excitotoxicity, and neuroinflammation. While many other stressors are shared by other cell types, including ER stress, amino acid deprivation, hypoxia, heat shock, viral infection and oxidative stress, their impact on neurons with specialized morphology and functions is not always comparable to that on other cells and tissues. Neurons therefore have specialized stress responses, which may involve LPS.

### Neuronal RNA is susceptible to oxidative damage

Oxidative stress, an imbalance between reactive oxygen species and antioxidant, is considered to be one of the major threats to neuronal survival in the CNS. Calcium signalling, glutamate uptake, high ATP demand, the importance of redox reactions, and low endogenous antioxidant defence in neurons all contribute to the neuronal vulnerability to oxidative stress [277], but the engagement of RNA oxidation in neurodegenerative diseases has been appreciated only recently.

Similar to proteins and DNA, RNA suffers oxidative damage. In fact, it is even more susceptible to oxidation than other cellular components [278,279], due to its storage in the form of membraneless RNP granules, resulting in its direct exposure to cytoplasm, where thousands of other chemical reactions take place, and due to its single-strandedness, which means it provides accessible sites for oxidative enzymatic reactions [278,279].

RNA oxidation can be functional, as it helps to break down damaged RNA in healthy cells [280], but can also compromise translation. Oxidatively damaged RNAs are altered structurally and are translated less efficiently owing to an increased frequency of ribosome stalling because of the failure in ribosome quality control [281]. Furthermore, the overall RNA levels including rRNA and tRNA, are significantly lower upon RNA oxidation, leading to compromised ribosome functioning and reduced availability of mRNA for translation in affected brain areas [282,283]. The consequences of translation attenuation resulting from RNA oxidative stress may be even more severe in axons and dendrites, where local translation takes place. In developing axons, a large proportion of RNA granules were found to localize adjacent to mitochondria as a major source of reactive oxygen species [179,284]. Moreover, neurites and synapses host activities associated with high metabolic rates and oxidative stresses, such as synaptic transmission.

Unsurprisingly, excessive RNA oxidative damage is associated with neurological disorders, mostly independent of genetic inheritance [285]. A high level of RNA oxidation has been detected in brains of AD, PD, and ALS patients, even preceding the development of pathological hallmarks like protein aggregation [286,287,288,289,290]. Furthermore, oxidative damage to RNA increases with aging due to progressive accumulation of free radicals that exceeds the capability of anti-oxidant defences, possibly accounting for the functional decline in aging brains and late onset of many neurodegenerative diseases [291,292]. However, there is currently insufficient evidence to determine whether RNA oxidative damage is disease-causative or a consequence of disease [287].

The compromised activity of the antioxidant enzyme superoxide dismutase 1 (SOD1), responsible for removing superoxide anions, is associated with multiple diseases, highlighting the importance of antioxidative defence system in neuronal health and survival [293,294,295]. Neurons expressing a pathogenic SOD1 mutant show defective axonal transport, distinct axonal transcriptomes and altered mitochondrial morphology and distribution along axons [53,296]. Intriguingly, oxidative stress is found to decrease RBP solubility through cysteine oxidation and to promote the formation of neuronal aggregates, such as stress granules [297]. RBP–RNA interactions may also be weakened due to RNA oxidative damage and RBP structural alterations, potentially enhancing RBP aggregation propensity. Consistently, the addition of mutant SOD1 aggregates effectively triggered the cytoplasmic aggregation of another ALS-associated protein, TDP-43 [298]. As discussed, the tight control of RBP solubility and cytoplasmic viscosity is key to axonal transport and LPS, which plays an important role in axonal mitochondrial functions and axon survival. Therefore, changes in axonal trafficking and the axonal transcriptome, together with perturbations of mitochondrial integrity in SOD1 mutant axons, point towards a hypothesis that SOD1 mutations are associated with impaired axonal protein synthesis, due to the failure of neuronal antioxidative defence.

### Neurons form stress granules with distinct properties in response to stress

*De novo* formation of translationally repressed stress granules (SGs) with diameters of 100 nm to 2 µm is widely observed upon exposure to a range of stressors and across an extensive range of cell types. Historically, the term ‘stress granule’ refers to cytoplasmic RNP granules containing polyadenylated RNA and certain ‘SG markers’, including poly(A)-binding protein (PABP), T cell intracellular antigen 1 (TIA-1), TIA-1-related protein (TIAR) and Ras GTPase-activating protein-binding protein 1 (G3BP1) [299,300]. During stress, RBPs present in SGs may selectively recruit mRNA targets to protect them from degradation, as demonstrated for Zipcode-binding protein 1 (ZBP1) [301]. In addition, mRNA deadenylation, which often precedes mRNA degradation, appears to be inhibited in SGs, implying a connection exists between SGs and RNA stability [302].

Formation of SGs occurs when translation initiation is limited by stress-induced eIF2α phosphorylation, resulting in local accumulation of mRNAs, translation initiation factors, small ribosomal subunits, and associated RBPs [299,303]. Facilitated by the ability of IDD-containing RBPs to phase separate, these factors coalesce into a compact structure, which serves as a stable SG ‘core’ to recruit other SG components as a more dynamic SG ‘shell’ [304]. It is an open question whether classic SG markers like TIA-1 and G3BP act as scaffolding proteins in the SG core or as shuttling components in the shell [304–306]. However, depletion of G3BP1 to inhibit SG formation did not seem to abolish stress-induced translation repression [307], nor did it accelerate mRNA degradation [305], suggesting that the accumulation of SG marker-containing SGs may be a consequence rather than a prerequisite for of cellular stress responses.

In narrow neuronal processes, accumulation of large SGs can pose a great risk to cargo transport and local proteostasis. In addition to SGs acting as ‘roadblocks’, mRNAs and translational machinery may be sequestered by stable SGs from their cytoplasmic pool, disengaging them from mRNA translation. For instance, axonal G3BP1-associated SGs have been shown to act as a negative modulator of LPS by sequestering a subset of mRNAs [308]. In cultured primary neurons, TDP-43/FUS-containing RNP granules are evident in axons in which aggregation-prone FUS mutants or FUS with altered PTMs are present, resulting in perturbed mRNA localization and LPS [56,58]. It is widely accepted that hyper-stable, amyloid-like deposits resulting from chronic stress in neurons are pathological hallmarks of neurodegenerative disorders [304,309,310], and pharmacological inhibition of SG formation and accumulation has been shown to delay neurodegenerative disease progression [311,312]. Therefore, understanding the role played by SG-modulated LPS during disease development may provide further insights into LPS-based therapeutic treatments.

Since SG formation is dispensable for activating the stress response yet may negatively impact on LPS-supported neuronal function, it is possible that neurons strategically prevent the formation of large rigid SGs during the stress response. Efforts to reveal the differences between acute stress-induced RNP granules and pathological aggregates have identified common components, especially RBPs, the mutations and aberrant PTMs of which are disease-relevant [309,313], suggesting a shared molecular origin between early SGs and pathological assemblies. Intriguingly, the formation and expansion of neuronal SGs are reported to be delayed and slow over the prolonged course of neurodegenerative diseases [57,314,315], in contrast to the rapid appearance of SGs in other cell types under stress [316]. This suggests specific factors are in place in neurons to control SG maturation. Indeed, a study combining proximity labelling and mass spectrometry revealed a large population of neuron-specific SG proteins, including neurodegeneration-associated proteins ELAVL2/3/4 [317]. Furthermore, SGs in neurites show different protein compositions compared to somal SGs, suggesting SGs may participate in compartment-specific activities. Notably, chaperones involved in protein folding and transport, as well as autophagy factors, are among the top-ranked neuronal SG proteins [317]. Chaperones have been shown to interact with stress granules to regulate their dynamic assembly and disassembly [318] and their role in clearing pathological aggregates is increasingly being appreciated in neurodegenerative disease studies [319,320].

### Neurons utilize compartmentalized stress responses to cope with stress

As long-lived cells, neurons incapable of coping with cellular stresses can come to suffer from chronic stress due to the accumulation of subtle stress-triggered alterations over years, which ultimately can lead to catastrophic consequences. Therefore, neurons must adopt various strategies to cope with distinct stresses.

A cellular stress response that is used widely by neurons as well as other cell types is the unfolded protein response (UPR). The UPR is activated to reduce the misfolded protein load when misfolded proteins come to accumulate in the ER, a process known as ER stress. The first cellular response to alleviate ER stress is to minimize further protein synthesis, which is mediated by the protein kinase RNA-like endoplasmic reticulum kinase (PERK) pathway. Essentially, upon UPR activation, PERK proteins, which are the transmembrane protein kinases of the pancreatic eIF-2α kinase (PEK) family, oligomerize and autophosphorylate. PERK also phosphorylates eIF2α, a component of the ternary translation initiation complex (which consists of eIF2, initiator methionine transfer RNA and guanosine triphosphate (GTP)). p-eIF2α decreases the availability of the ternary complex and thus global protein synthesis by inhibiting the activity of the guanine exchange factor eIF2B, which is responsible for loading GTP onto the ternary complex after each round of translation initiation [321]. Paradoxically, certain mRNAs escape such translation repression and are instead translated more efficiently upon eIF2α phosphorylation, facilitated by upstream open reading frames located at the 5ʹUTR of their mRNAs. One such mRNA is that encoding activating transcription factor 4 (ATF4), which activates the transcription of pro-apoptotic gene CCAAT-enhancer-binding protein homologous protein (CHOP). Protein synthesis repression caused by UPR activation is associated with a wide range of neurodegenerative disorders, including AD, PD, and prion diseases, and restoration of translation activity is neuroprotective in disease models [322].

While the signalling pathway resembles that found in other cell types, the neuronal UPR features the spatiotemporal segregation of specific components, resulting in a compartmentalized stress response unique to neurons. For instance, in a study in which hippocampal axons were exposed to AD-associated peptide Aβ_1-42_, axonal p-eIF2α levels increased, indicating UPR activation. Unexpectedly, in contrast to the canonical stress response that results in global translational repression, axonal protein synthesis was significantly increased, including axonal ATF4 synthesis. Over the next 24 hours, ATF4 was retrogradely transported to the soma, where it activated CHOP-dependent apoptosis and led to neuron death [323]. The authors demonstrated that inhibition of local synthesis of ATF4 or its retrograde transport upon axonal Aβ_1-42_ treatment could effectively reverse CHOP activation and cell loss, exemplifying a form of inter-compartmental signalling propagation in neurodegenerative diseases.

Interestingly, while activation of the UPR is extensively associated with human diseases, the pathway itself has evolved to be a robust pro-survival pathway to mitigate cellular stress in adverse situations, particularly when the insult is mild and transient [324]. The UPR also has various physiological functions, such as protein quality control and metabolism [325,326]. Neurons also use the UPR or individual components of the pathway to regulate physiological activities in the absence of classical stress or pathology [327,328]. In developing retinal ganglion cell axons, the increase in LPS upon 10 min of stimulation by the guidance cue Semaphorin 3A (Sema3A) is partly mediated by the PERK pathway [329]. Sema3A stimulation induces PERK activation and eIF2α phosphorylation, but similar to the Aβ_1-42_-induced response, axonal protein synthesis is also significantly increased. Therefore, it has been proposed that this differential outcome of eIF2α phosphorylation can be explained by Sema3A stimulation eliciting rapid local synthesis and dephosphorylation of eIF2B, generating a higher level of ternary complexes for translation initiation [329]. This unique Sema3A-induced PERK activation in axons provides a first insight into how neurons engage a modified stress response to meet their developmental demands.

## Conclusion and further perspectives

In both neurodevelopmental and neurodegenerative disorders, dysfunction of axons and synapses has been proposed to be central to the observed pathology. Neurodevelopmental disorders like FXS and ASD result from failure in the establishment of synaptic connectivity [330]. In contrast, in neurodegenerative disorders, such as AD, Huntington’s disease and prion diseases, synapse loss is among the first pathological signs, and the extent of synapse loss is the best correlate for cognitive decline [331,332,333]. In the case of ALS, the ‘dying-back model’ has been proposed, in which loss of the axon and motor neuron innervation is initiated in the distal compartment [334]. Encouragingly, it has been reported for several animal models of neurological disorders that synaptic dysfunction and concurrent cognitive impairments are reversible during neurodevelopment and at the early stage of neurodegenerative diseases [335,336,337,338], making research into the underlying mechanisms that compromise synapse integrity highly attractive for therapeutic development.

In this review, we have discussed evidence that LPS in neurites is critical to neuronal function, and that it is compromised in neurological disorders. As LPS supports the autonomy of distal compartments, both through the support of homeostasis and as a localizable regulatory response mediator, its dysregulation particularly affects neuritic maintenance and function. Expectedly, failure in LPS regulation may directly contribute to the neurite dysfunction found in many neurological disorders. However, it should be borne in mind that LPS deficiency can also be downstream of disruption in other processes key to neurite survival, such as axonal trafficking. Therefore, a major challenge to thoroughly understanding the role of LPS in neuropathy is to elucidate the causal relationship between LPS perturbation and various disease-associated pathophysiology.

It is not always straightforward to prove an alteration in LPS rather than somatic translation accounts for a disease phenotype. In recent years, several methods have been developed to perform unbiased screens for axonally synthesized proteins in culture [339]: both laser-capture microdissection [10,340] and compartmentalized culture systems, such as modified Boyden Chambers [39,43,341], allow for axon-only samples to be collected. Similarly, microfluidic devices enable the spatial separation of neuronal cell bodies and axons into fluidic isolated compartments connected by 150–600 µm long microgrooves. This not only allows the somatodendritic and axonal material to be collected separately, but also enables specific manipulations to be performed on the axonal compartment without affecting the soma, including methods that selectively label axonal mRNAs and proteins or inhibit mRNA translation locally [44,342]. However, trafficking between the axonal and somal compartments makes this kind of compartmentalized culture experiment less reliable for the investigation of processes that occur on timescales of days. Furthermore, these systems do not recapitulate the range of cues observed in the *in vivo* context, for instance during synapse formation, which may be important regulators of LPS. These challenges mean the role of compromised LPS in synapse formation and maintenance in neurological disorders is still largely unknown, and further technical advances are being developed to address this.

Subcellular *in vivo* multi-omics technology has emerged in the past few years as a method of choice to elucidate the role of LPS in the interconnected neuronal context of animal models of disease, as shown by three recent studies. The first two of these studies employed the RiboTag (also known as axon-TRAP) system to identify cell-type-specific ribosome-bound mRNAs in axons [9,152]. The neurons chosen in these studies, RGCs and auditory cortical TE3 neurons, have their axons and somas situated at spatially distinct locations, which can therefore be surgically separated *in vivo*. As revealed by the RiboTag approach, the repertoire of ribosome-associated mRNAs in mouse RGC axons changes with the developmental stage to support various functional requirements during axon development and maintenance [9]. The study in auditory cortical axons showed that the translatome was altered during the consolidation of associative memory, for instance with mitochondrion-related genes being upregulated and cytoskeleton-related genes being downregulated [152]. In the third study, a method for determining the transcriptome and proteome of growth cones of selectively labelled neurons was developed: *in vivo* fluorescent labelling of callosal protein neurons of only one hemisphere through *in utero* electroporation, allowed purification of trans-hemispheric growth cones, by homogenization of the appropriate hemisphere, subcellular fractionation, and use of a modified fluorescence-activated cell sorting setup. This allowed comparison of different neuronal subtypes and highlighted the molecular specialization of the growth cone, where both the mTOR kinase protein and mRNAs containing mTOR-dependent motifs were accumulated [343]. Furthermore, labelling of nascent proteomes *in vivo* can be achieved by cell-type-specific metabolic labelling using a methionine analogue, azidonorleucine [344,345,346]. Although it is yet to be applied to study the axonal compartment, this technical procedure has shown great compatibility with surgical separation of subcellular compartments *in vivo*. Assisted by these powerful *in vivo* methods, similar comparisons of the local translatome in disease models and healthy animals at different developmental stages would provide further insight into the extent to which LPS is disrupted in neurological disorders.

To fully establish a causative link between LPS and neurological disorders, however, methods for *in vivo* local inhibition of LPS will need to be developed. So far, it has been successfully demonstrated for *Xenopus* retinal projection that the local introduction of mRNA-specific anti-sense oligonucleotides (morpholinos) can inhibit local mRNA translation [14,116]. However, *in vivo* manipulation of axonal translation is more technically challenging in less accessible mammalian neurons. Surgical exposure of axon bundles in live animals followed by local compound treatment or dye labelling is sometimes possible for certain peripheral neurons, such as the sciatic nerve in the hind limb [347]. Excitingly, the past decade has witnessed the rapid development of novel optogenetic approaches for neuroscience research conducted on small mammals *in vivo* [348]. Meanwhile, elegant optogenetic tools to manipulate intracellular organelle positioning [349], protein phase states [350] and translational activities [351] have been designed and refined to yield new discoveries with high spatiotemporal precisions. All these technical advances in optogenetics, although yet to be tested, hold great promise for facilitating the investigation of LPS in animal models *in vivo*.

In addition to further investigating the complex regulation of axonal LPS in the *in vivo* context, the role of LPS in other neuronal compartments and non-neuronal cells should also be considered. We have used the axon as an example of the ways LPS can support distal compartments, as it is the most of a highly polarized neurite, but it should be noted that LPS also supports some unique functionalities of dendrites that are disrupted in neurological disorders. For instance, LPS is associated with long-term depression triggered by metabotropic glutamate receptors in dendrites. Loss of FMRP protein enhances this response, resulting in altered synaptic plasticity [352]. Furthermore, there are also other unique features of neuronal tissues that can create unique vulnerabilities, to disruption of LPS as well as to other insults. In particular, neuronal connectivity has here been simply taken to give rise to unique functional requirements that are supported by LPS and compromised in neurological disorders, but the interconnected nature of neurons itself can be a source of vulnerability in some disorders. In neurodegenerative diseases that are associated with protein aggregation, aggregates often the first form in particular regions of the brain, and then ‘spread’ through a characteristic sequence of other brain areas in a prion-like manner, which mirrors the brain’s internal connectivity [353]. Additionally, there is also ample evidence that the function of non-neuronal cells is compromised in neurological disorders, which affects neuronal function, and can again be linked to LPS in some cases. LPS occurs in non-immune glial cells (astrocytes and oligodendrocytes), where it is known to be important to cell function and health, and LPS of key proteins in protrusions of glial cells has found to be reduced in ALS [354]. Furthermore, stresses originated in non-neuronal cell types can strongly affect neuronal cell populations and neurite homoeostasis. Stress within glia themselves may also be detrimental to neuronal survival, as has been shown for activation of the unfolded protein response in astrocytes [355]. Another notable example of such a stress is neuroinflammation: activation of microglia following neuronal damage can result in proinflammatory signalling that can result in neuronal death in several ways [356]. Excitotoxicity due to excessive glutamate signalling is another stress that is associated with signalling between neurons as well as glia: it can occur through astrocyte dysfunction, and is associated with neurodegenerative diseases as well as ischemic stroke [357].

As a final note, this review has limited itself to neurological disorders for which there is an identifiable genetic basis, allowing disease models to be developed relatively easily, and thus does not reflect the full variety of neurological disorders. Some sporadic neurodegenerative cases may be associated with a range of interacting genetic risk factors of low penetrance, or with exposure to environmental factors, or both, and model systems in which these factors can to an extent be replicated would be very informative. Furthermore, some neurological disorders can clearly be considered to be ‘acquired’, such as following traumatic injury, which can be more readily replicated in experimental systems. Intriguingly, for example, it has been shown for substance addiction that LPS and its upstream signalling networks are affected by the altered activity of microRNA networks [358] and specific RNA-binding proteins [359]. It would be interesting to consider the similarities and differences between LPS in these different forms of neurological disorders.
